# Three-Dimensional Printing Surgical Applications

**Published:** 2015-08-14

**Authors:** Ahmad B. AlAli, Michelle F. Griffin, Peter E. Butler

**Affiliations:** ^a^Centre for Nanotechnology & Regenerative Medicine, UCL Division of Surgery & Interventional Science, University College London, London, United Kingdom; ^b^Department of Plastic and Reconstructive Surgery, Royal Free London NHS Foundation Trust Hospital, London, United Kingdom

**Keywords:** 3D printing medical and surgical applications, medical education using 3D printing, 3D printing prosthesis, surgical planning using 3D printing, 3D printed surgical instruments

## Abstract

**Introduction:** Three-dimensional printing, a technology used for decades in the industrial field, gains a lot of attention in the medical field for its potential benefits. With advancement of desktop printers, this technology is accessible and a lot of research is going on in the medical field. **Objective:** To evaluate its application in surgical field, which may include but not limited to surgical planning, surgical education, implants, and prosthesis, which are the focus of this review. **Methods:** Research was conducted by searching PubMed, Web of science, and other reliable sources. We included original articles and excluded articles based on animals, those more than 10 years old, and those not in English. These articles were evaluated, and relevant studies were included in this review. **Discussion:** Three-dimensional printing shows a potential benefit in surgical application. Printed implants were used in patient in a few cases and show successful results; however, longer follow-up and more trials are needed. Surgical and medical education is believed to be more efficient with this technology than the current practice. Printed surgical instrument and surgical planning are also believed to improve with three-dimensional printing. **Conclusion:** Three-dimensional printing can be a very powerful tool in the near future, which can aid the medical field that is facing a lot of challenges and obstacles. However, despite the reported results, further research on larger samples and analytical measurements should be conducted to ensure this technology's impact on the practice.

Three-dimensional (3D) printing is a method whereby an actual structure is created by layering technique using the computer-aided design software, which relays the signals to the 3D printer.^1^ This notion was initially presented in 1984 by Chuck Hull when he designed a process known as stereolithography. This process consisted of adding layers one after another over the top of each other, using curing photopolymers along with UV lasers.^2^ After 1984, this process began to gain momentum and received appreciation worldwide and held importance in different fields, including the medical field. The growing market for 3D desktop printers gives rise to extensive research in that field; medical application may include surgical planning, prosthesis, implanted structure, medical education, and other applications.^3^ The most attractive feature of 3D printing is that custom-made items can be produced, which are valuable for the patients as well as the surgeons. For instance, surgeons will be able to use 3D printing instead of organ transplantation. Organ transplant is a very expensive procedure, and it is difficult to find a donor match and even then many a times it results in organ rejection. In cases of emergency, it also becomes very difficult to arrange for an organ that will be compatible with the recipient's body system. In year 2009, more than 150,000 patients alone in the United States were on the waiting list of organ transplantation. However, only 27,996 patients received a transplant, which comprises only 18% of the patients of the original donor list. Many others who did not receive a transplant died of organ failure. The numbers in the previous year (2014) have increased, but only a small percentage of those patients receive required organs. Hence, 3D printing will use cells of the patients and build an organ that can replace the old, diseased one. Moreover, 3D printing has a vital role in neurosurgery where cranial implants are required. Patients with a head injury require fitting of a cranial plate. 3D printing is being used to produce the accurate design and fitting of the cranial plates.^4^

In this review, we aim to discuss the current application of 3D printing in the surgical field, along with the future perspective of this tool and how it affects surgical practice. 3D printing is indeed making its way in the surgical departments of hospitals, and many surgeons are using this technology, but on a small-scale 3D printing has shown high prospects of becoming a technology that can eradicate the use of transplanting organs from one person to another as well as reduce common issues such as sterilization that occur during surgical procedures.

Since first introduced, 3D printers have undergone huge developments in the past 3 decades. Different types of 3D printers are manufactured, depending on the uses, materials, and accuracy. Recently, a range of developers and companies urge to market the concept of desktop 3D printers, branding it as the new personal computer revolution. Since then, a huge development in the field has attracted consumers with a highly competitive atmosphere between all developers, medium-sized companies, and huge ones.^5^ Although desktop printers ranging from home-assembled parts cost about $200 to $3000, the professional 3D printer can be very expensive and can cost up to more than $500,000,000. In Table 1, we aim to introduce a quick comparison between the most prominent 3D printer types, how they works, advantages, disadvantages, and materials of each printer for better understanding of the 3D printing process.^6^

## METHODS

**Figure 1:**
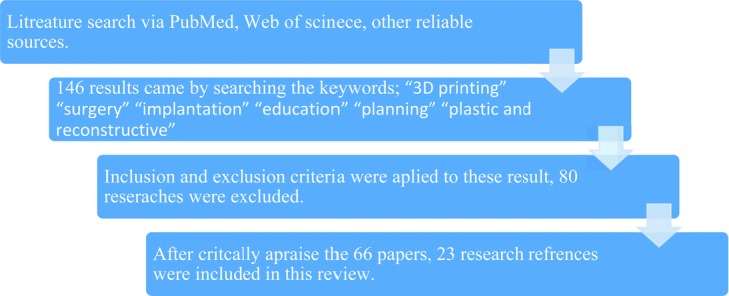
Methodology of this literature review; stepwise approach.

Literature was examined via PubMed, Web of science, and other reliable sources using key words “3D printing,” “surgery,” “implantation,” “education,” “planning,” and “plastic and reconstructive.” A total of 146 research articles were obtained. The criteria for exclusion were research conducted in languages other than English, articles that were not from this decade (10 years), and research articles whose full text could not be accessed. Moreover, the criterion for inclusion of articles was that they should be the original research. As a result, 80 research articles were excluded and the remaining left were 66 studies that were assessed. After critical assessment, 23 of these 66 references were included in this article (Fig 1).

## RESULT

### Surgical planning

Before initiating a surgery, it is extremely vital for the whole surgical team to collaborate for an effective surgery, the fundamental of those are that there should be comprehensive understanding of the condition of the patient and how to apply the surgical knowledge in the right manner. Surgical planning has achieved a lot of improvement with the ongoing progress of the imaging procedures that give the surgeons better visualization and better understanding for the anatomy and pathology of the patient.^7^ While the current practice might be adequate for surgical planning in some cases, other complex cases required better understanding that will lead to better results, decreased incidence of complications, and decreased time of surgical procedures. 3D printing possesses the ability to produce similar type of models of body organs and structures. This has generated a lot of interest in the field of surgical planning among different surgical specialties. Different centers around the world present cases in which a fabricated model was created from the imaging modalities using computer-aided design, which was then printed with a 3D printer, followed by the planning procedure by the treating team.^8^^-^10

#### Cardiac surgery

In a case presentation of a 70-year-old patient with extensive arteriosclerotic aneurysm reaching from the ascending aorta to the descending aorta, the team decided to print a 3D model to evaluate how this can help surgery option decision, as the case was complicated and the comorbidities included arterial hypertension and diabetes mellitus. 3D model was fabricated resembling the patient's heart from computed tomographic (CT) scans. A decision was made to undertake the frozen elephant trunk procedure, and the treating team clearly stated that the model helped them take the decision and also facilitated planning depth and diameter of the stent as well as the landing zone in the patient. Although it might not be necessarily the case for each surgery, it aided the planning in this complex case.^8^^-^10

Other study was about 2 patients on the heart transplantation list due to failing single ventricle repair and medical and surgical interventions that were unsuccessful. The first patient was a 2-year-old boy with failing staged palliation of hypoplastic left heart syndrome. Despite the surgical intervention, he suffered from severe tricuspid regurgitation and heart failure. The second patient was a 14-year-old girl with pulmonary atresia and hypoplastic right ventricle. Shunt was performed in the first month of her life, followed by a central shunt. She was doing well till she was admitted with signs of severe protein-losing enteropathy, and she was listed for heart transplantation as well. 3D model of both patients’ hearts was fabricated with an inkjet printer and it was sterilized and taken into the surgical theater. The surgeon had the chance to develop the optimal approach and to anticipate the problems that might arise, especially with the complex anatomy of the cases. As they underwent more than 1 surgery in the past, the team had the information about the special requirements and dimensions of the donor heart, including the measurements of the inferior vena cava, pulmonary artery, and aortic arch. Thus, it can be concluded that the use of 3D printing provided the surgeon with practical advantages of demonstrating exact anatomy of the patient prior to the surgery.^11^

#### Neurosurgery

In the field of neurosurgery, planning is very important as the area the surgeon is dealing with is very sensitive. 3D printing is also a part of this planning, and neurosurgeons show interest in this tool. In a case presentation of 2 patients with lesions in the proximity of the motor cortex, a 3D model was fabricated prior to the surgery from 3-Tesla magnetic resonance imaging (MRI). The accuracy of these printed models was estimated both theoretically and by simulated phantom experiments with physical measurements and repeated MRI. 3D models gave the surgeons additional information about the entry point, which may help in avoiding damage to areas of eloquent cortex. Moreover, the model gave a clear vision of both depth and extension of the tumor. The models can also improve communication with the patient.^12^

#### General surgery

Small tumors in the liver that are candidate for surgery require adequate understanding of the structure, vascular branches, and the decision that determine the area that will be resected. The cases of 2 patients with synchronous multiple liver metastases in segments 2, 6, and 7 from rectal cancer are presented. After chemotherapy, the tumor in segment 2 was invisible by ultrasonography. Multiple detector CT was performed and a 3D image was created to fabricate a 3D model using stereolithography. After the production, the surface was covered with support material, which was washed later on and washed and coated with resin paint. The plan was carried out using that model, which was segmentectomy of segment 2 with concomitant partial resection of segment 3 and posterior sectionectomy with concomitant resection of the dorsal segment of the anterior sector. Resection was done successfully, and the result was confirmed by a negative histological margin. Printing of the model made the procedure easy and feasible. It could also be a good alternative, especially when dealing with small tumors, as the ultrasonography might be misleading because of the probe angle, which may give different views of the vessels. However, the cost of printing of 70% scaled liver was approximately US $420, and production that took 18 hours to print may be the limitations that need improvement.^13^

#### Plastic surgery

In plastic and reconstructive surgery, a good understanding of the defect and anatomical relations is crucial to achieve better excision and reconstruction. The case of an 82-year-old patient who underwent elective ankle replacement surgery complicated by wound dehiscence, infection, and exposed prosthesis is presented. After serial debridement failure, the patient was scheduled for soft-tissue coverage with dead space filling, reconstruction with radial forearm free flap was planned, CT-angiography was performed, and a 3D model was printed that enhanced the understanding of the defect morphology and enabled hands-on preoperative planning. Another study includes 10 patients with different case presentations, all candidates for osteoplastic flap surgery. 3D model was used to produce an onlay template of the frontal sinus and an adjacent area was created using CT images, with frontal sinus margins accurately up to a 5-mm range maximum. This method was found to be consistently accurate, osteoplastic flap margin within 1 mm of the actual frontal sinus margin, although there are no data to compare this with the current mapping modalities. 3D model printing is likely more precise than the 2-dimensional images such as CT of frontal sinus models and onlay templates in osteoplastic flap surgery.^14^

From previous studies along with other studies shown in Tables 2–4, it is clear that a lot of research and trials are going on for 3D printing in the field of surgery. We discuss these study limitations and future perspectives in the “Discussion” section.

### Surgical instruments printing

Surgical instrument prices and management are vital issues in the surgical field, with advancement in 3D printing, which is a new era of printing. These instruments may have a potential benefit on different aspects; printing instruments that can be specific to the patient, reduction in the prices of the instruments that are used currently, and the availability of instruments in less developed countries and areas as well as in war zones. In a study, a navy retractor was printed using 3D technology by an FDM printer, levels of sterility were tested, and mechanical power of the instrument was also checked. It tolerated 11.3 ± 0.57 kg and broke at 15.9 ± 0.8 kg, printing time was only 90 minutes, 1 kg of polylactic acid or polylactide material cost $27 and can produce 61 costume retractors, which is 1/10 compared with the current stainless steel. The instrument developed possesses the strength that is required in the operation theater and can be a source of readily available sterile surgical instruments. However, because of inaccessibility of the 3D printing machinery all over the world, it is not yet a suitable technique in the underdeveloped or developing countries.^15^

### Surgical education

Surgical residents’ exposure to different cases along with understanding the exact anatomical features helps better education environment. It is also important at the level of medical students to understand different pathologies and anatomical variations demonstrated in a clear manner. Recently, 3D printing was also an area of interest in that field to help better understand patients’ conditions and anatomical variations on a 3D model rather than 3D image on a flat 2-dimensional screen. In a study, a 3D model of portal veins and hepatic veins was created by current-selective laser-sintering technique using CT and MRI. The cost was less than $100 per model. These models are patient-specific whereby they can be used for teaching residents and medical students in surgical intervention and these can also be used as physical models that are easier than imaging modalities that need knowledge for interpretation. As dissection is more beneficial than passive observation, the example of hands-on experience may be applied here too.^16^

**Figure 2:**
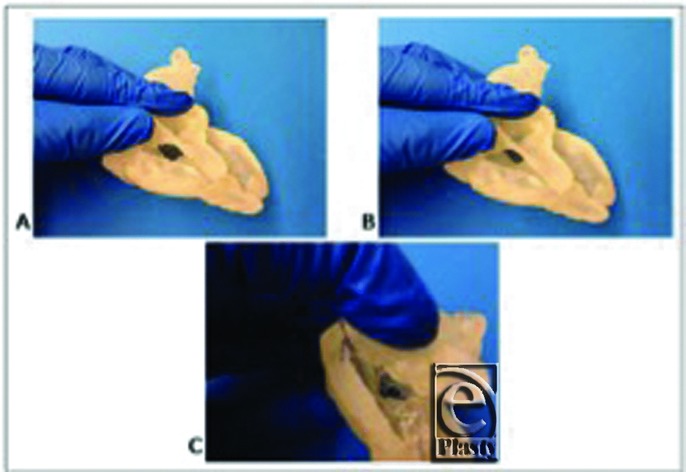
Three-dimensional models for different type of ventricular septal defect. From Costello et al.^17^

Another example is printing anomalies in the heart-like ventricular septal defect. In a study, a model of each type of the 5 common ventricular septal defects was fabricated with a polyjet 3D printer using MRI (Fig 2). This module was demonstrated in a session of students and a pre- and postsession assessment questionnaire was given, the results of which were as follows: the knowledge acquisition result on a scale of 1 to 10 was 3.22 before the seminar and 7.02 after the seminar, knowledge reporting was 2.16 before the seminar and 6.60 after the seminar, and structural conceptualization was 2.17 before the seminar and 6.31 after the seminar, (*P* < .0001).^17^

### 3D printing for implants

Alloplastic materials are now being preferred because of their advantages in the medical field. Unless designed so, synthetic material does not resorb with time, giving a permanent and long-lasting look. They can be customized according to the requirement of the patient and also reduce operating time, as graft from another site of the patient's body is not required. There are several types of alloplastic materials such as silicone, polymethyl methacrylate, polyamide mesh, and others. The type of surgical procedure to be performed and the character of the injury or defect being augmented are the factors that often influence the selection of alloplastic materials to be used. Implants are very important in medicine. In various cases, structures need to be replaced or repaired by an implant; in some cases, the implants are not available to manufactures or are not suitable for patient-specific cases.^18^

**Figure 3:**
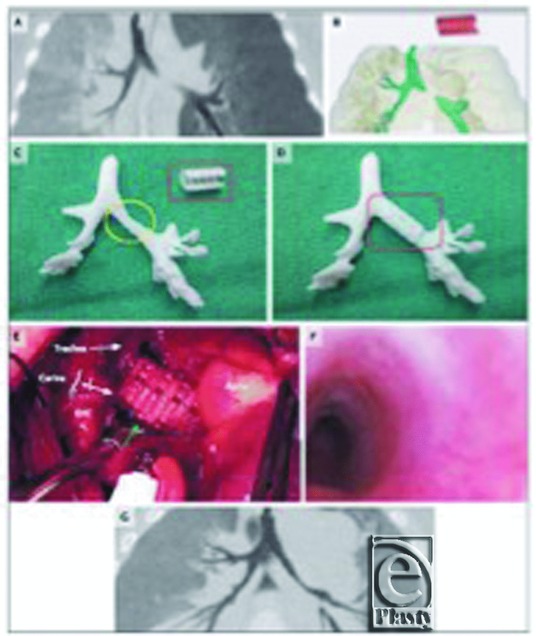
Three-dimensional printed airway splint. From Zopf et al.^19^

In a study, successful implant of an airway stent was performed for a child born with tracheobronchomalacia who started to have chest retraction at the age of 6 weeks but progressive deterioration happened at the age of 2 months and required endotracheal intubation, but ventilation to prevent the cardiopulmonary arrests could not be maintained. A custom-fabricated polycaprolactone respiratory tract splint was developed using a 3D printer to provide resistance against collapse and to allow simultaneously flexion, extension, and expansion with growth (Fig 3). The Food and Drug Administration permitted the use of the implant with the exemption of use only in emergency. The implant was inserted and bronchoscopy was performed that revealed normal patency of the bronchus without collapse. Twenty-one days after the procedure, ventilation was discontinued, and 1 year postoperatively, follow-up tests and endoscopy showed left patent main stem bronchus and no other complication was seen.^19^

In another case presentation of patients with non–weight-bearing maxillofacial deformities as a result of congenital anomalies, trauma and tumor resection were discussed. Reconstruction was carried out by using 3D printing technology and produced an “inkjet-printed custom-made artificial bone” (IPCAB) for both cases in this study. A 55-year-old woman with postsurgical facial deformity who 20 years back had undergone a partial resection of tongue and segmental osteotomy of the mandible. Reconstruction was performed with skin flap and autologous costochondral graft preceded by autologous iliac bone graft due to resorption of costochondral graft. Despite these treatments, face deformity persisted and she was a candidate for the IPCAB. Another case was that of a 23-year-old man with a deviation of the mandible with malocclusion, which was treated by bilateral mandibular distraction and le Forte type I osteotomy. Eighteen month later, this patient underwent mandible reconstruction and genioplasty with IPCAB.^20^

## DISCUSSION

The application of 3D printing has gained momentum in the medical field, although limitations in this field are present and it is becoming an easy and accessible tool since the desktop 3D printers were introduced with an aim to be a part of every home and institution.^21^ As discussed in the Introduction, different 3D printing technologies are available, with advantages and disadvantages of each. Most of these printers are being evaluated by researchers in the medical field to be applied in practice. As discussed earlier, 3D printing is a tool that might aid in different ways and be an added value for the surgical field. Its benefits include surgical planning, medical education, patient education, implants, prosthesis, and other applications.

In the field of surgical planning, numerous attempts in different fields to understand the pathologies and complex anatomical structure are undertaken by different centers, comparing the current practice that depend on imaging with the fabricating 3D printing models for better hands-on experience and surgical planning and education. However, studies should be conducted before creating a clear deduction that this technology is compulsory for cases such as these. Moreover, in most of these cases, a general statement was given regarding the improved surgical preparation with the lack of an accurate method of measuring this and outcomes of these cases compared with the current practice.^2^

Concerning medical schooling, all studies we have reviewed discussed resident education and improved understanding of the intricate structure and potential exercise on that structure before the real surgical procedure. This may be a useful tool in the upcoming time that requires questioning and researching, addressing the rare cases, complicated cases, and stimulate training on these 3D models prior to the surgery. Although surgeons have started using implants, a further study is needed before it develops into a common procedure or practice. A detailed follow-up is required to assess these cases and complications that might rise on the long term. Although it seems as a promising field in the upcoming years, ethical issues should be paid attention to while running the trials to guarantee no harm is caused to the patients.^1^

On the contrary, 3D printing has given rise to certain safety concerns, as 3D printing can be used for criminal and illegal purposes. In theory, it is possible that this technology can be used to produce counterfeit medical devices and instruments, medicinal drugs, and other such items. Hence, its usage will require strict regulation from the authorities.^22^ Moreover, another factor that can impede the usage and development of 3D printing technology in the medical field is the approval from the Food and Drug Administration. It might seem as a hurdle for mass scale production by 3D printing.^23^

While we are yet to reach the level where 3D printing becomes a common practice in surgery, a promising field of 3D printing applications is in organogenesis, customized nutritional products, and drugs, which may be the future of medicine and surgery. Being able to print a highly complex organ or in situ printing, that is, tissue embedded with cells is implanted in the patient during surgery, is an aspect of 3D printing that might take 20 years or more to fully develop, but the progression is promising and it may become a valid modality in the future.^4^

## CONCLUSION

3D printing is a promising era that may have a huge influence on the field of medicine. A wide-ranging study is necessary on large samples and over a long period to address possible advantages of 3D printing in surgical procedures. It plays a certain role in manufacturing implants and prosthetics for patients, and 3D printing also plays a vital role in planning and training for surgical procedures as well as other aspects of medical education.

## RECOMMENDATION

To study the exact benefit of 3D printing in resident teaching, surgical planning, and medical education, as well as other potential benefits.

## Figures and Tables

**Table 1:** Different types of 3D printing machines*

Type	Mechanism	Advantages	Disadvantages	Material	Other
SLA	UV light is used to create the object by curing and solidifying a liquid resin	• High-resolution prototypes • Good finishing at the surface	• Support structure is needed • Require postcuring	Resin; a curable laser photopolymer or other plastic-like products	Layer thickness: 0.05-0.2 mm
SLS	Laser fuses the layers of a powder material	• Ability to produce complex/functional parts • Does not require support structure • High productivity	• Surface finish is rough	Plastic, nylon, polystyrene, metals; steel, titanium, and composites	Layer thickness: 0.06-0.18 mm
FDM	Extruding small beads of the melted plastic material, which hardens afterward	• No postcuring • Easy material changeover • Low-cost machines compared with others	• Slow processing, especially on large parts • Low detailing accuracy • Low surface finish integrity	Filament of thermoplastic polymer; ABS, PLA	Layer thickness: 0.15-0.25 mm (adjustable)
DLP	DLP projector projects the light in a repetitive process	• Good resolution • Fast processing time	• Support structure is needed	Liquid resin	Accuracy: 139 μm
INKJET	Spraying liquid or the photopolymer depending on the type of the jetting; binder or material	• A variety of material choice • High precision • Colored parts	• Require postcuring	Plastic, metal, and ceramics	Layer thickness: material: 0.013 mm (min) Binder: 0.09 mm (min)

*From Protosys^5^ and Horvath.^6^ 3D indicates 3-dimensional; ABS, acrylonitrile butadiene styrene; PLA, polylactic acid or polylactide.

**Table 2:** Surgical planning using 3D printing*

Study title	Authors	Journal	Objective of the study	Year	Result	Conclusion
Three-Dimensional Printing for Perioperative Planning of Complex Aortic Arch Surgery	Schmauss et al	*The Annals of Thoracic Surgery*	• Evaluate the use of fabricated 3D model in surgical planning for a 70-year-old patient referred for complete aortic arch replacement. After being diagnosed with extensive arteriosclerotic aneurysm reaching from the ascending aorta to the descending aorta, patient's comorbidities include arterial hypertension and diabetes mellitus.	2014	• Preoperative 3D model facilitated decision-making process to perform a complex and high-risk FET procedure • The model facilitated planning the depth and diameter of the stent as well as the landing zone in the patient • Not every patient undergoing aortic arch surgery is a candidate for fabrication of stereolithographic models	Future studies on a larger number of patients expected to show that stereolithography facilitates preoperative planning and decreases the risk of complex aortic arch surgery
Paediatric Cardiac Transplantation: Three-Dimensional Printing of Anatomic Models for Surgical Planning of Heart Transplantation in Patients With Univentricular Heart	Sodian et al	*The Journal of Thoracic and Cardiovascular Surgery*	• Evaluate the use of fabricated 3D model in surgical planning for 2 complicated cardiac pediatric cases for heart transplantation	2008	3D model: • Helps develop the optimal surgical approach using the 3D fabricated models • Aids in anticipation of the problems that might be faced by the surgeon • Facilitates a better understanding of the complex anatomy after patients underwent multiple surgical procedures	The use of 3D printing provided the surgeon with practical advantages of demonstrating exact anatomy of the patient prior to the surgery
Three-Dimensional Printing of Models for Preoperative Planning and Simulation of Transcatheter Valve Replacement	Schmauss et al	*The Annals of Thoracic Surgery*	Evaluate the use of fabricated 3D model in surgical planning for transcatheter valve replacement	2012	The use of 3D model: • Maximizes surgical anticipation of problems • May facilitate the decision concerning the size of the TAVI prosthesis • Provides the exact position of the crucial structure	Use of the model not expected to change the basic surgical plan. Although it helped in understanding that extremely calcified aortic root and the sinus of Valsalva became small, inelastic, and stiff, positive results may be achieved by deep implantation without occluding the whole sinus of Valsalva’
Three-Dimensional Printing of Models for Surgical Planning in Patients With Primary Cardiac Tumors	Schmauss et al	*The Journal of Thoracic and Cardiovascular Surgery*	Evaluate the use of fabricated 3D model in surgical planning in cardiac tumor surgery	2013	• 3D models may be helpful tool for preoperative decision planning of surgical intervention lead to a better understanding of position and infiltration of the cardiac tumor into cardiac tissue, especially when imaging modalities such as MRI, CT, and echocardiography are insufficient	3D printing model provides surgeons and interventionists with both theoretical and practical advantages in treating complex pathology in cardiac surgery
Preoperative Three-Dimensional Model Creation of Magnetic Resonance Brain Images As a Tool to Assist Neurosurgical Planning	Spottiswoode et al	*Stereotactic and Functional Neurosurgery*	Evaluate the use of fabricated 3D model in surgical planning for 2 cases of patients with lesions in the proximity of the motor cortex	2013	3D models: • Gives the surgeons additional information can affect the entry point to avoid • Gives a clear vision of both; depth and extension of the tumor • Improves communication with the patient	3D models’ accuracy for brain surgery was acceptable, with a mean dimensional error of 0.5 mm, and this may be good tool for surgical training and planning
Application of a Three-Dimensional Print of a Liver in Hepatectomy for Small Tumours Invisible by Intraoperative Ultrasonography: Preliminary Experience	Igami et al	*World Journal of Surgery*	Evaluate the use of fabricated 3D model in surgical planning for 2 cases of patients with synchronous multiple liver metastases small tumors after receiving chemotherapy and compare that to ultrasonography	2014	• The surgery plan was carried out using 3D printing model • 3D printing may be a good alternative for ultrasonography as it might be misleading due to probe handling	3D printed models for small tumors aid the surgery and made it easy and feasible; however cost reduction, speed of production and automation of the post production steps are the current limitations
3D Printing Haptic “Reverse” Model for Preoperative Planning in Soft Tissue Reconstruction; A Case Report	Chae et al	*Microsurgery*	Evaluate the use of fabricated 3D model in surgical planning for soft tissue reconstruction	2014	• 3D model enhances the understanding of the defect morphology • The model helps in preoperative planning	Surgical outcomes might be improved using 3D models compared with current 2D imaging modalities
Frontal Sinus Models and Onlay Templates in Osteoplastic Flap Surgery	Daniel et al	*The Journal of Laryngology & Otology*	Evaluate the use of fabricated 3D model in surgery for frontal sinus mapping	2011	• These models are consistently accurate to within 1 mm • 3D model printing is likely more precise than the images showing in 2D such as CT	3D models can be used intraoperatively as an onlay guide for frontal sinus mapping; however, comparison with other mapping techniques is needed

*From Chae et al,^7^ Schmauss et al,^8^^-^10 Sodian et al,^11^ Spottiswoode et al,^12^ Igami et al,^13^ and Daniel et al.^14^ 3D indicates 3-dimensional; CT, computed tomography; FET, frozen elephant trunk; MRI, magnetic resonance imaging; 2D, 2-dimensional.

**Table 3:** 3D printing in surgical education*

Study title	Authors	Journal	Objective of the study	Year	Result	Conclusion
A Low-Cost Surgical Application of Additive Fabrication	Watson	*Journal of Surgical Education*	Evaluate the use of 3D printing models in surgical education for residents and medical students	2014	• Patient-specific 3D models can be used to teach the residents and medical students on the surgical intervention • As a physical model, it is unlike CT and MRI; does not need imaging interpretation skills • Hands-on experience superior to observation	3D models may have a value in education applications, with a great potential in intra-abdominal and intrathoracic anatomical and surgical education
Utilizing Three-Dimensional Printing Technology to Assess the Feasibility of High-Fidelity Synthetic Ventricular Septal Defect Models for Simulation in Medical Education	Costello et al	*World Journal for Pediatric and Congenital Heart Surgery*	Evaluate the use of 3D printing models in medical education	2014	• Knowledge acquisition, knowledge reporting, and structural conceptualization significantly improved after the session with 3D models of different common VSD types	3D printing is a feasible modality to be used in medical education and it is a foundation for stimulation-based education

*From Watson^16^ and Costello et al.^17^ 3D indicates three-dimensional; CT, computed tomography; MRI, magnetic resonance imaging; VSD, ventricular septal defect.

**Table 4:** 3D printed surgical implants*

Study title	Authors	Journal	Objective of the study	Year	Result	Conclusion
Bioresorbable Airway Splint Created With a Three-Dimensional Printer	Zopf et al	*The New England Journal of Medicine*	Present the experience of implanted airway stent for a child born with tracheobronchomalacia	2013	• The subsequent bronchoscopy revealed normal patency of the bronchus without collapse • Ventilation was discounted 21 d after the surgery • Follow-up imaging and endoscopy showed patent left mainstem bronchus; no complications were observed 1 y after stent implantation	3D fabricated models created using high-resolution imaging and computer-aided design; can produce implantable patient-specific devices
Maxillofacial Reconstruction Using Custom-Made Artificial Bones Fabricated by Inkjet Printing Technology	Saijo et al	*Journal of Artificial Organs*	Evaluate the use of IPCAB in reconstruction surgery.	2009	• Patient satisfaction was achieved by reconstruction using IPCAB • There was no severe local or systemic reverse event • Union observed as early as 6 mo and in 12 mo in 1 patient • IPCAB achieved the mechanical strength sufficient for surgical handling	IPCAB is safe to be used, and it achieved compatibility with good biodegradability and osteoconductivity; long-term follow-up is needed

*From Zopf et al^19^ and Saijo et al.^20^ 3D indicates three-dimensional; IPCAB, inkjet-printed custom-made artificial bone.
